# CCL28 promotes locomotor recovery after spinal cord injury via recruiting regulatory T cells

**DOI:** 10.18632/aging.102239

**Published:** 2019-09-26

**Authors:** Pengfei Wang, Xiangbei Qi, Guohui Xu, Jianning Liu, Jichao Guo, Xu Li, Xinzhe Ma, Hui Sun

**Affiliations:** 1 Department of Neurosurgery, The Third Hospital, Hebei Medical University, Shijiazhuang 050051, China; 2 Department of Orthopaedics, The Third Hospital, Hebei Medical University, Shijiazhuang 050051, China

**Keywords:** CCL28, CCR10, spinal cord injury, locomotor recovery, regulatory T cell

## Abstract

Background: Chemokines play a key role in post-traumatic inflammation and secondary injury after spinal cord injury (SCI). CCL28, the chemokine CC-chemokine ligand 28, is involved in the epithelial and mucosal immunity. However, whether CCL28 participates in the physiopathologic processes after SCI remains unclear.

Results: CCL28 is upregulated in the spinal cord after SCI. In addition, neutralizing antibodies against IL-1β or TNF-α, or treatment of ML120B, a selective inhibitor of IKK-β, remarkably decrease CCL28 upregulation, suggesting that CCL28 upregulation relies on NF-κB pathway activated by IL-1β and TNF-α after SCI. Moreover, CD4^+^CD25^+^FOXP3^+^ regulatory T (Treg) cells that express CCR10, a receptor of CCL28, are enriched in the spinal cord after SCI. We further demonstrate that the spinal cord recruits Treg cells through CCL28-CCR10 axis, which in turn function to suppress immune response and promote locomotor recovery after SCI. In contrast, neutralizing CCL28 or CCR10 reduces Treg cell recruitment and delays locomotor recovery.

Methods: The neutralizing antibodies and recombinant CCL28 were injected intraspinally into the mice prior to SCI, which was established via hemitransection. RT-qPCR analysis was performed to determine transcript level, and Western blot analysis and ELISA assay were used to detect protein expression. Immune cells were analyzed by flow cytometry and visualized by immunofluorescence. The chemotaxis was assessed by *in vitro* transwell migration assay. The mouse locomotor activity was assessed via the Basso Mouse Scale (BMS) system.

Conclusions: These results indicate that NF-κB pathway-regulated CCL28 production plays a protective role after SCI through recruiting CCR10-expressing and immunosuppressive Treg cells, and suggest that interfering CCL28-CCR10 axis might be of potential clinical benefit in improving SCI recovery.

## INTRODUCTION

Spinal cord injury (SCI) often causes a multifaceted spectrum of complex posttraumatic sequelae endangering the whole neuraxis of patients, such as paralysis and loss of locomotivity [1]. SCI is generally characterized by primary injury derived from mechanical trauma and secondary injury induced by a plethora of cellular and molecular pathological changes, including inflammatory response, vascular damage, excitotoxicity, apoptosis and metabolic disorder, etc., in which the inflammatory response in the spinal cord is deemed to play a central role and acts synergistically with these secondary injury mechanisms to worsen the clinical outcome, such as reducing the prospect for neurological recovery [2–5]. Thus, interfering or manipulating the processes and mechanisms of secondary damage could serve as an important strategy for SCI therapy [6]. Nonetheless, at present, the effective treatment used for eliminating the secondary damage is still lacking.

The inflammatory response after SCI is orchestrated by a variety of cell types, including astrocytes, microglia, endotheliocytes and infiltrating immune cells [7]. It has been demonstrated that the infiltration of immune cells is a major contributor to the secondary damage after SCI [3]. Following an increase in the permeability of blood-spinal cord barrier (BSB) after mechanical damage [8], the blood monocytes, granulocytes and lymphocytes migrate across the defective BSB and enter into the injured spinal cord, where they act together with a strong expression of inflammatory mediators to induce the inflammatory response in tissue surrounding the original injury sites [9]. This inflammatory response may persist for days or weeks and ultimately leads to degeneration of myelin, neuronal apoptosis and scar formation, thus deteriorating neurological dysfunction [10].

In addition, it is known that immune cells are recruited to the injured sites by chemokines, and some studies have shown that several chemokines are upregulated within few minutes and hours after SCI, such as CCL2 [11], CCL20 [12], CCL3, CCL5, CXCL2/3 and CXCL10 [13]. These observations point to an important role of chemokines in mediating inflammatory cell recruitment. Moreover, neutralization of CXCL10 or CCL20 improves the neurological outcome after SCI [12, 14, 15], and chemokine antagonist also promotes axonal sparing in the rat SCI model [16], suggesting that chemokines and their cognate receptors may serve as possible targets in the modulation of the secondary damage after SCI [17].

CCL28 (the chemokine CC-chemokine ligand 28), also called mucosae-associated epithelial chemokine (MEC), is secreted from mucosal epithelial cells and involved in mucosal immunity [18]. CCL28 is a ligand for two chemokine receptors including CCR10 and CCR3, and its production can be induced by pro-inflammatory cytokines [19], indicating that it may play a role in attracting CCR10^+^ and/or CCR3^+^ cells to inflammatory sites. In truth, previous studies have demonstrated that CCL28 can exert chemotactic and immunomodulatory activities in inflammation and infection by recruiting CCR10^+^ IgA or IgE antibody-secreting cells [20], T regulatory (Treg) cells [21, 22], and mucosal T and B cells [23, 24]. These features of CCL28 suggest it may serve as an anchoring factor bridging the innate and adaptive immunity [25]. Beyond conventional recognition, CCL28 expression has also been convinced in the central nerve system [18]. And in a rat model, CCR10 gradually increased in a time-dependent manner after SCI [26]. Moreover, Ccl28 was identified as a node of network of differentially expressed genes in mice with SCI [27]. However, to date, the regulation, function and underlying mechanisms of CCL28 itself after SCI are poorly understood. In the present study, we report the NF-κB pathway-upregulated production of CCL28 in the spinal cord after SCI, and identify the positive role of CCL28 in promoting locomotor recovery after SCI through recruiting CCR10-expressing and immunosuppressive Treg cells via CCL28-CCR10 axis, thereby revealing a novel role of CCL28 in regulating the secondary damage after SCI.

## RESULTS

### CCL28 is upregulated in the spinal cord after SCI

**Figure 1:**
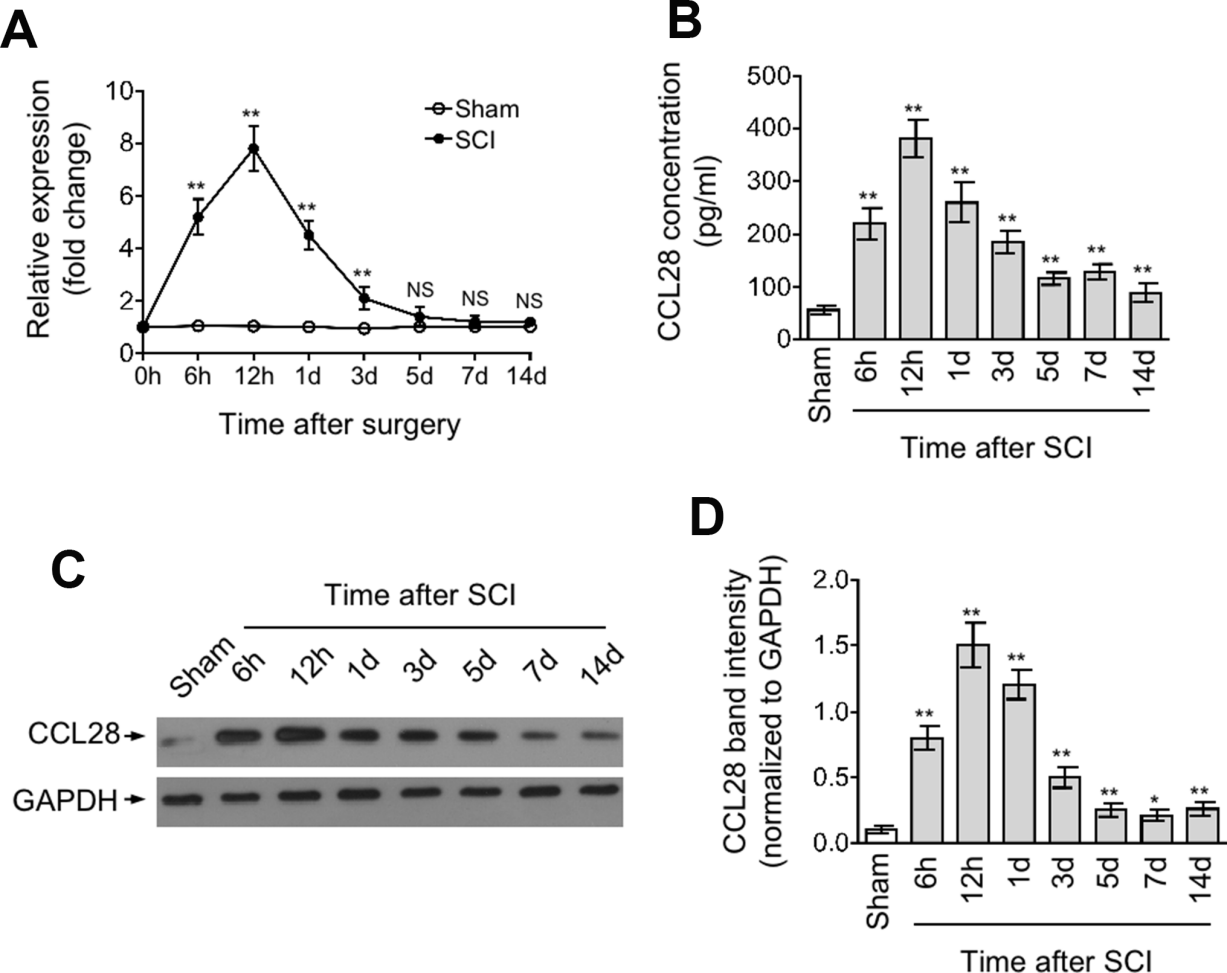
**CCL28 is upregulated in the spinal cord after SCI.** (**A**, **D**) qRT-PCR analysis of CCL28 mRNA level (A), ELISA analysis of CCL28 protein concentration (**B**), and Western blotting analysis of CCL28 protein expression (**C**) and band intensity analysis of CCL28 (**D**) in the spinal cord at different time points after sham or SCI surgery (n=5). GAPDH was used as a reference or loading control. Data are mean ± SD. Data were compared with sham group and statistical analysis was performed using Student’s *t*-test. **, P<0.01; *, P<0.05; NS, not significant.

We hypothesized that if CCL28 associates with the secondary damage or even plays a role after SCI, it may exhibit expression change during the pathogenic processes after injury. To test this possibility, we first measured the mRNA level of CCL28 in the spinal cord at different time points from 0 hr to 14 days after SCI by qRT-PCR analysis. As shown, in SCI group, compared with that at 0 hr, the mRNA level of CCL28 in the spinal cord was promptly increased within multiple hrs and peaked nearly by 8-fold at 12 hrs, but then gradually declined to the basal level at 5 days after SCI (Figure 1A, upper line). In contrast, the mRNA level of CCL28 remained almost steady in sham group (Figure 1A, lower line). Next, to examine whether the production of CCL28 is also increased in the spinal cord after SCI, we determined its protein level through ELISA and Western blot analyses. Consistent with the results depicted in Figure 1A, these results showed that CCL28 production in the spinal cord was also increased after SCI, and the only difference was that its protein level still remained higher than that of sham group at 5 days thereafter (Figure 1B–1D). These lines of evidence indicate that the expression of CCL28 in the spinal cord responds to injury after SCI by upregulation, particularly at the early phase, which exhibits substantial resolution at later phase, but with its protein amount remaining higher than the physiological level within a relatively long period, implying a corresponding duration for exerting its biological activities.

### IL-1β and TNF-α upregulate CCL28 through activating NF-κB after SCI

**Figure 2:**
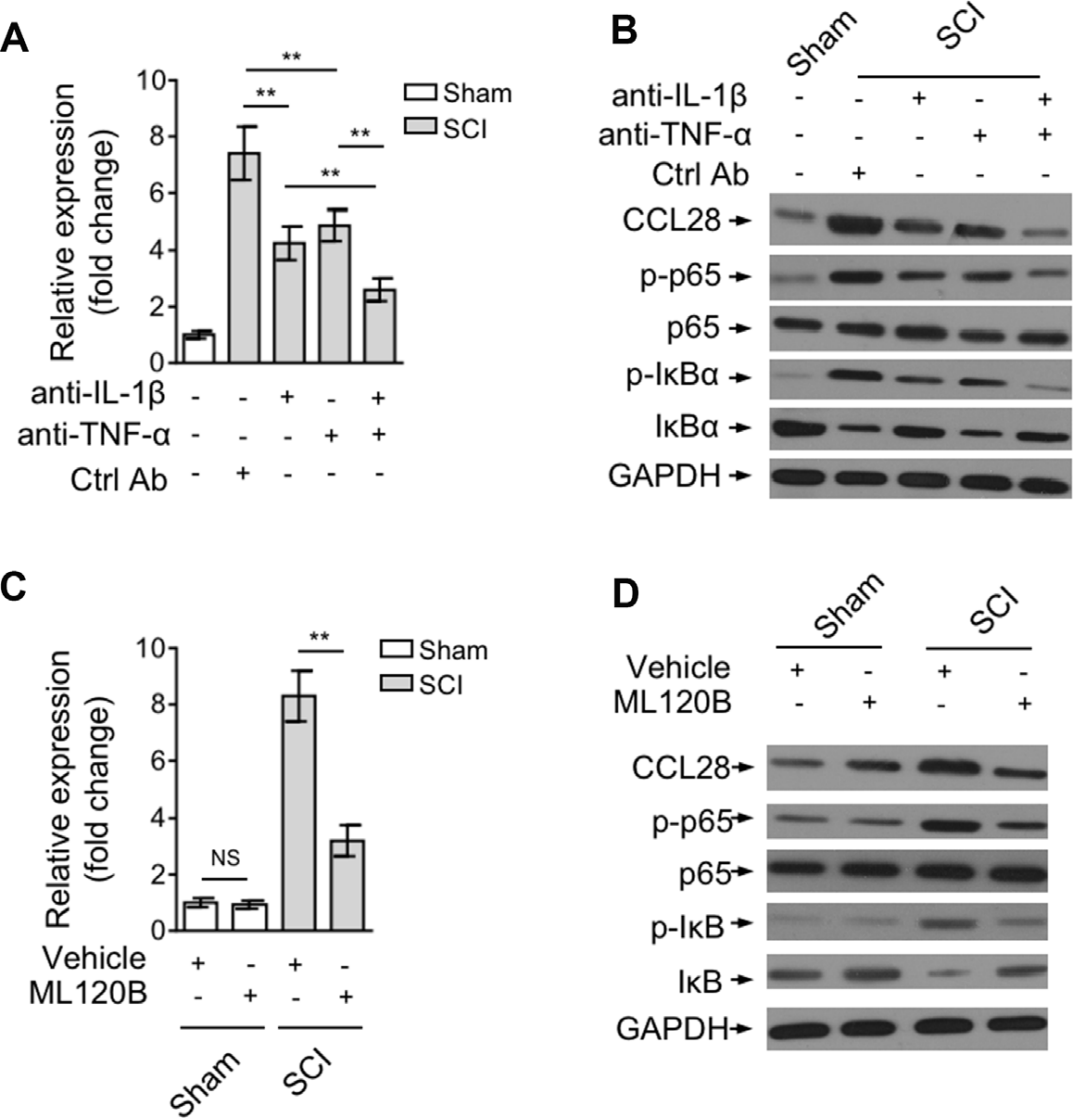
**IL-1β and TNF-α upregulate CCL28 through activating NF-κB after SCI.** (**A**, **B**) Mice were pre-injected with control antibody (Ctrl Ab) or neutralizing antibodies against IL-1β (anti-IL-1β) and/or TNF-α (anti-TNF-α) into the intraspinal cord for 12 hrs, and then subjected to sham or SCI surgery. After another 12 hrs, the spinal cord samples were analyzed by qRT-PCR and Western blotting for detecting CCL28 mRNA level (**A**) and protein level (**B**) (n=5). (**C**, **D**) Mice were pre-injected with equal volume of vehicle or 60 mg/kg ML120B into the intraspinal cord for 12 hrs, and then subjected to sham or SCI surgery. After another 12 hrs, the CCL28 mRNA level (**C**) and protein level (**D**) in the spinal cord was analyzed as in (**A**, **B**) (n=5). GAPDH was used as a reference or loading control. Data are mean ± SD. The statistical analysis was performed using Student’s *t*-test. **, P<0.01; NS, not significant.

The production of CCL28 has been associated with inflammation [22, 28–30] In airway epithelial cells, IL-1β and TNF-α could induce its expression via an NF-κB-dependent manner [31]. In addition, NF-κB pathway is activated after SCI, which is tightly controlled by proinflammatory cytokines, and in turn NF-κB plays a vital role in mediating inflammatory responses [32, 33]. To understand how CCL28 in the spinal cord is upregulated after SCI, we tested whether inflammatory cytokine-regulated NF-κB pathway is involved in this process. For this purpose, we blocked IL-1β and/or TNF-α in the spinal cord via intraspinally pre-injecting neutralizing antibodies into SCI mice. qRT-PCR analysis showed that the increased mRNA level of CCL28 in the spinal cord at 12 hrs after SCI was attenuated when IL-1β or TNF-α was blocked by neutralizing antibodies, which was more remarkable when they were blocked both (Figure 2A). The NF-κB pathway was indeed activated after SCI, as evidenced by the increased expression of phosphorylation of p65 and IκBα, which was, however, substantially inhibited when IL-1β and/or TNF-α were blocked (Figure 2B). In agreement with the change of its mRNA level (Figure 2A) and NF-κB status, the upregulated protein level of CCL28 in the spinal cord after SCI was also largely recovered when administrated with neutralizing antibodies against IL-1β and TNF-α (Figure 2B). These observations suggest that the expression of CCL28 is positively associated with NF-κB activation induced by proinflammatory cytokines, i.e., IL-1β and TNF-α.

To establish a direct link between CCL28 upregulation and NF-κB activation after SCI, we administrated SCI mice with ML120B, one potent and specific small molecular inhibitor of IKKβ, to inhibit NF-κB pathway [34]. The result showed that the mRNA level (Figure 2C) and protein level (Figure 2D) of CCL28 in the spinal cord after SCI was overtly decreased when NF-κB pathway was inhibited by ML120B treatment (Figure 2D), although not totally recovered to that of mice in sham group. Anyhow, these findings point to a critical role of inflammatory cytokine-stimulated NF-κB activation in upregulating CCL28 expression after SCI.

### CCR10-expressing CD4^+^CD25^+^FOXP3^+^ Treg cells are enriched in the spinal cord after SCI

**Figure 3:**
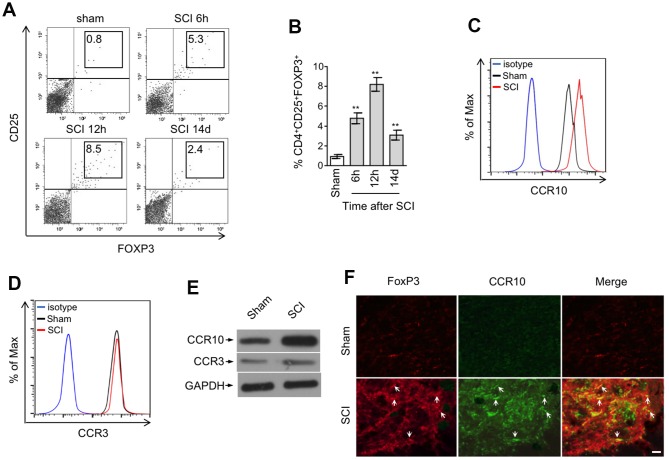
**CCR10-expressing CD4^+^CD25^+^FOXP3^+^ Treg cells are enriched in the spinal cord after SCI.** (**A**, **B**) Representative flow cytometry dot plots (**A**) and percentage (**B**) of CD4^+^CD25^+^FOXP3^+^ cells among the CD4^+^ T cells in the spinal cord after sham or SCI surgery. Cells were gated on 7-AAD negative population and the numbers inside plots refer to % Treg cells (n=5). (**C, D**) Representative histogram of CCR10 (**C**) and CCR3 (**D**) expression in CD4^+^CD25^+^FOXP3^+^ cells as gated in (**A**). (**E**) CD4^+^CD25^+^FOXP3^+^ cells in the spinal cord from sham and SCI mice were sorted out and analyzed by Western blotting to detect the expression of CCR10 and CCR3. GAPDH was used as a loading control. (**F**) Double immunostaining of FOXP3 (red) and CCR10 (green) in the spinal cord sections after sham or SCI surgery. The merged images are also shown. Arrows indicate cells with positive staining and colocalization. Scale bar, 100 μm. Data are mean ± SD. The statistical analysis was performed using Student’s *t*-test. **, P<0.01.

Previous studies have revealed that CCL28 shows chemotactic activity toward CCR10-expressing regulatory T (Treg) cells under inflamed or hypoxic condition [21, 22, 35]. In view of the upregulation of CCL28 in the spinal cord after SCI, we therefore examined whether Treg cells are recruited to the injured spinal cord. The frequency of CD4^+^CD25^+^FOXP3^+^ Treg cells within the population of CD4^+^ cells in the spinal cord at representative time points after SCI was determined by flow cytometry analysis. As shown, compared with sham group, the frequency of Treg cells was increased at 6 hrs after SCI, peaking at 12 hrs and then declining thereafter (Figure 3A, 3B). Coincidentally, this tendency is similar to the production change of CCL28 in the spinal cord after SCI (Figure 1B–1D), implying that CCL28 may be associated with the enhanced recruitment of Treg cells to the injured spinal cord. CCR3 and CCR10 were identified as two receptors for CCL28 [18, 36]. Intriguingly, FACS analysis showed that compared with Treg cells in the spinal cord of sham mice, CCR10 (Figure 3C), but not CCR3 (Figure 3D), was upregulated in the recruited Treg cells in the spinal cord of SCI mice. Moreover, this was further validated by Western blotting analysis showing that CCR3 was barely detected, however CCR10 was more abundantly expressed by the recruited Treg cells in the spinal cord after SCI (Figure 3E). These data suggest that CCR10 may be more functionally important in the recruitment of Treg cells after SCI. Furthermore, the enhanced recruitment of CCR10-expressing Treg cells in the spinal cord after SCI was confirmed by the co-localization of marker proteins FOXP3 and CCR10 (Figure 3F). Hence, our evidence demonstrates that CCR10-expressing Treg cells are enriched in the spinal cord after SCI

### Spinal cord recruits Treg cells through CCL28-CCR10 axis after SCI

**Figure 4:**
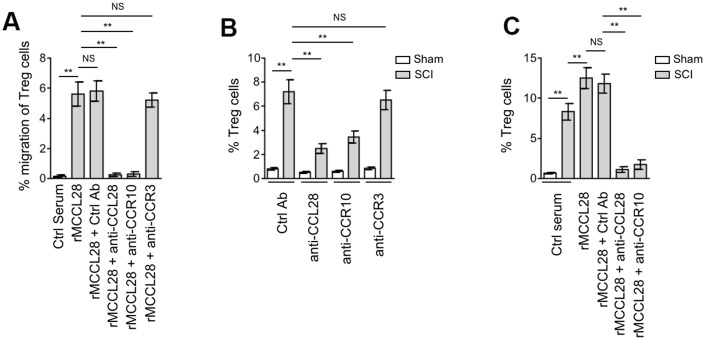
**Spinal cord recruits Treg cells through CCL28-CCR10 axis after SCI.** (**A**) Mouse peripheral blood mononuclear cells (PBMCs) were seeded in the upper chambers and pretreated with control antibody (Ctrl Ab), neutralizing antibodies against CCL28, CCR10 or CCR3 for 1 hr. The percentage of CD4^+^CD25^+^FOXP3^+^ Treg cells among the CD4^+^ cells recruited to the lower chambers with medium containing mouse recombinant CCL28 (rMCCL28) or 1% mouse control serum was analyzed by flow cytometry (n=6 replicates in each group). (**B**) Mice were pre-injected with Ctrl Ab or neutralizing antibodies against CCL28 (anti-CCL28), CCR10 (anti-CCR10) or CCR3 (anti-CCR3) into the intraspinal cord for 12 hrs, and then subjected to sham or SCI surgery. After another 12 hrs, the percentage of CD4^+^CD25^+^FOXP3^+^ Treg cells in the spinal cord was determined by flow cytometry analysis (n=5). (**C**) Mice were pre-injected with Ctrl Ab, anti-CCL28 or anti-CCR10 and rMCCL28 or 1% mouse control serum as indicated into the intraspinal cord for 12 hrs, and then subjected to sham or SCI surgery. After another 12 hrs, the percentage of CD4^+^CD25^+^FOXP3^+^ Treg cells in the spinal cord were determined (n=5). Data are mean ± SD. The statistical analysis was performed using Student’s *t*-test. **, P<0.01; NS, not significant.

To elucidate whether spinal cord recruits CCR10-expressing Treg cells via CCL28, we first tested the chemotactic activity of mouse recombinant CCL28 (rMCCL28) towards freshly isolated mouse peripheral blood mononuclear cells (PBMCs) using an *in vitro* chemotaxis assay. Indeed, compared with control serum, rMCCL28 supplementation recruited more Treg cells within PBMCs, which was totally abrogated by the pretreatment of neutralizing antibody against CCL28 or CCR10, as compared with control antibody (Figure 4A). However, only subtle effect was observed when CCR3 was blocked by neutralizing antibody (Figure 4A). To further investigate whether this also applies to the enhanced recruitment of Treg cells to the spinal cord after SCI, we intraspinally pre-injected neutralizing antibody against CCL28, CCR10 or CCR3 into the SCI mice. Similar to that observed in the *in vitro* chemotaxis assay, the result showed that the recruitment of Treg cells to the spinal cord was substantially decreased in SCI mice treated with CCL28 or CCR10 neutralizing antibody, and CCR3 neutralization had no similar effect (Figure 4B). Hence, these results suggest that CCL28 recruits Treg cells mainly through its binding to CCR10, both *in vitro* condition and in the spinal cord of SCI mice. The promoting effect of CCL28-CCR10 axis on Treg cell recruitment to the spinal cord of SCI mice was further substantiated by the evidence that rMCCL28 intraspinal administration enhanced Treg cell recruitment, which was abrogated when CCL28 or CCR10 was blocked by neutralizing antibody (Figure 4C). Therefore, these findings illustrate that the spinal cord recruits Treg cells via CCL28-CCR10 axis after SCI.

### CCL28 promotes locomotor recovery after SCI through recruiting Treg cells

**Figure 5:**
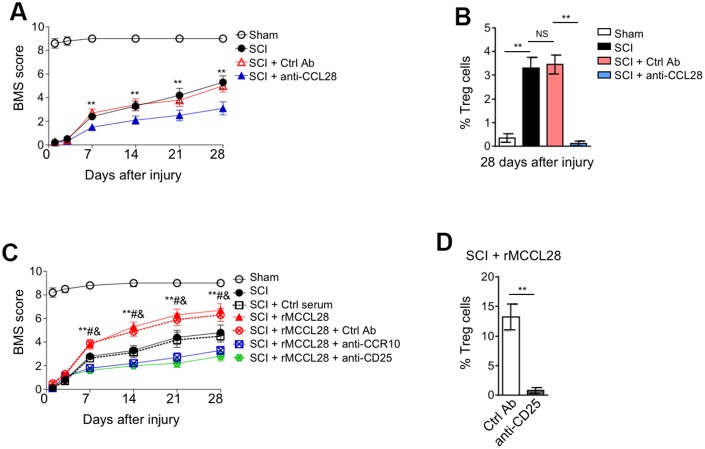
**CCL28 promotes locomotor recovery after SCI through recruiting Treg cells.** (**A**, **B**) Mice were pre-injected with Ctrl Ab or anti-CCL28 into the intraspinal cord for 12 hrs, and then subjected to sham or SCI surgery. The injection of Ctrl Ab or anti-CCL28 was repeated at 14 days after injury. The locomotion recovery was monitored using the BMS open-field test to determine locomotor capabilities (**A**) and the percentage of Treg cells at 28 days after injury was assessed by FACS analysis (n=8). Data are mean ± SEM. Data were analyzed by repeated measures analysis of variance (ANOVA). ** P<0.01 represents the comparison between SCI + Ctrl Ab group and SCI + anti-CCL28 group. (**C**) Mice were pre-injected with Ctrl Ab, anti-CCR10, anti-CD25, rMCCL28 or 1% mouse control serum as indicated into the intraspinal cord for 12 hrs, and then subjected to sham or SCI surgery. The injection of Ctrl Ab, anti-CCR10, anti-CD25, rMCCL28 or 1% mouse control serum was repeated at 14 days after injury. The locomotion recovery was monitored (n=8). Data are mean ± SEM. Data were analyzed by repeated measures analysis of variance (ANOVA). ** P<0.01 represents the comparison between SCI + Ctrl serum group and SCI + rMCCL28. # P<0.01 represents the comparison between SCI + rMCCL28 + Ctrl Ab group and SCI + rMCCL28 + anti-CCR10 group. & P<0.01 represents the comparison between SCI + rMCCL28 + Ctrl Ab group and SCI + rMCCL28 + anti-CD25. (**D**) The percentage of CD4^+^CD25^+^FOXP3^+^ Treg cells in the spinal cord from SCI + rMCCL28 + Ctrl Ab group and SCI + rMCCL28 + anti-CD25 group was determined (n=8). Data are mean ± SD. The statistical analysis was performed using Student’s *t*-test. **, P<0.01.

CCL28 is involved in immune regulation at various mucosal linings and displays broad-spectrum antimicrobial activity [37, 38]. We show here that CCL28 is upregulated in the spinal cord after SCI, to learn more about the role of CCL28 involved in this process, we tested whether it affects locomotor recovery after SCI. Determination of mouse locomotor activity in open field via the Basso Mouse Scale (BMS) system [54] showed that the recovery was significantly delayed in SCI mice when administrated with CCL28 neutralizing antibody, as compared with control antibody (Figure 5A, 5B), together with the previous results (Figure 4B, 4C), suggesting that the delayed locomotor recovery may be associated with the decreased Treg cell recruitment. To establish a direct link between CCL28-mediated Treg cell recruitment and locomotor recovery after SCI, SCI mice were treated with rMCCL28 in combination with neutralizing antibody against CCR10 or CD25 for either abrogating Treg cell recruitment or directly depleting CD25^+^ T cells. The results of BMS test showed that compared with control serum, rMCCL28 treatment promoted locomotor recovery after SCI, whereas, this effect was completely reversed when the recruitment of Treg cells into the spinal cord was impaired by the neutralization of CCR10 or CD25 (Figure 5C, 5D and Figure 4C). Thus, a direct causal link exists between CCL28 upregulation in the spinal cord and improved locomotor recovery, which can at least be attributed to Treg cell recruitment through CCL28-CCR10 axis.

### Treg cells mediate immune suppression in the spinal cord after SCI

**Figure 6:**
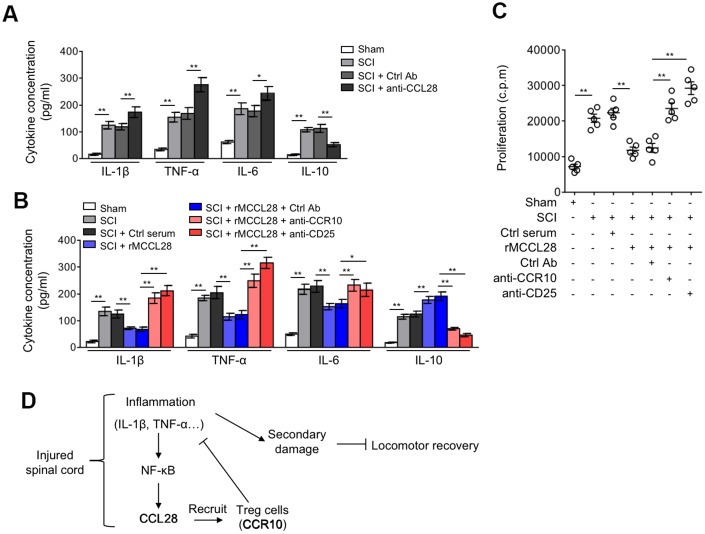
**Treg cells mediate immune suppression in the spinal cord after SCI.** (**A**) Mice were treated as in Figure 5A. ELISA analysis of cytokine concentration in the spinal cord at 7 days after sham or SCI surgery (n=5). (**B**, **C**) Mice were treated as in Figure 5B. (**B**) The cytokine concentration in the spinal cord at 7 days after sham or SCI surgery were determined as in (**A**) (n=5). (**C**) The proliferation rate of effector T cells was determined by [^3^H]-thymidine incorporation analysis (n=5). c.p.m., counts per minute of incorporated [^3^H]-thymidine. Data are mean ± SD. The statistical analysis was performed using Student’s *t*-test. **, P<0.01; *, P<0.05. (**D**) A brief schematic model of this study. After SCI, inflammatory cells infiltrate into the spinal cord and secrete cytokines, including IL-1β and TNF-α, which promptly induces the production of CCL28 via NF-κB activation. Responding to increased CCL28 in the focal sites, CCR10-expressing Treg cells are recruited and then exert their immune suppressive activities, restricting the inflammation to a controllable extent along with the time consumed. Owing to the activity of Treg cells recruited by CCL28, the local levels of IL-1β and TNF-α are decreased, thereby in turn relieving the stimulative effect on CCL28 upregulation, through this negative feed-back loop, CCL28 functions to suppress inflammation, reduce secondary damage and promotes locomotor recovery after SCI.

It’s well-established that inflammatory reaction contributes substantially to the secondary injury after SCI, and that its targeting improves functional recovery in SCI animal models [33, 39, 40]. Post-traumatic inflammation is coordinated by pro- and anti-inflammatory cytokines, in which IL-1β, TNF-α and IL-6 are proinflammatory cytokines critical for mediating the post-traumatic inflammatory reaction, whereas, IL-10 is a potent anti-inflammatory cytokine that suppresses the function of inflammatory cells [41, 42]. It’s also known that Treg cells play an indispensable role in maintaining immune tolerance and suppressing excessive immune reaction detrimental to the host [43, 44]. Therefore, to understand how the recruited Treg cells contribute to improved locomotor recovery after SCI, we focused on examining their immunosuppressive role in the spinal cord. We first assessed the influence of CCL28 neutralization on local inflammation by measuring the production of IL-1β, TNF-α, IL-6 and IL-10 at 7 days after SCI. We found that the protein expression of IL-1β, TNF-α and IL-6 was further increased, and oppositely, IL-10 was decreased in the spinal cord of SCI mice when administrated with CCL28 neutralizing antibody, as compared with control antibody (Figure 6A). These results indicate that the local inflammatory response in the spinal cord is promoted when CCL28 was neutralized. On the other side, compared with control serum, rMCCL28 treatment decreased the protein level of IL-1β, TNF-α and IL-6, and increased that of IL-10 (Figure 6B). Whereas, these effects were reversed when CCR10 or CD25 was neutralized (Figure 6B). Additionally, consistent with this more tolerogenic immune environment forged by rMCCL28 treatment, the effector T cells in the spinal cord of SCI mice showed less proliferation rate, which, however, was recovered by the neutralization of CCR10 or CD25 (Figure 6C). Overall, these data prove that Treg cells, recruited to the spinal cord by CCL28-CCR10 axis, mediate immune suppression in the spinal cord after SCI, thus at least in part accounting for the functional role of Treg cells in improving locomotor recovery in the mouse SCI model (Figure 6D).

## DISCUSSION

Accumulating evidence has demonstrated that the chemokine-ligand/receptor-network is a key element involved in the secondary lesion cascades induced by SCI, such as CCL20/CCR6 [12], CX3CL1/CX3CR1 [45] and CXCL10/CXCR3B [46]. In terms of factors which are capable of modulating inflammatory responses after SCI, the chemokine-ligand/receptor-network assumedly harbors promising targets for future mechanism-driven trials [47]. However, the interactive paradigm between secondary damage after SCI and this network is still not fully delineated. In the present study, we for the first time discovered that a novel chemokine CCL28 was upregulated in the spinal cord after SCI (particularly at early phase) in both mRNA and protein levels, and that this upregulation initiating very likely at the transcriptional level gradually vanished at later phase. We suspect that this temporal change pattern of CCL28 expression is probably due to the resolution of inflammation during recovery after SCI, because we and others have shown that the expression of CCL28 is closely related to inflammation and NF-κB pathway activation induced by proinflammatory cytokines *in vitro* and *in vivo*, such as IL-1β and TNF-α [19, 31]. This negative feedback regulation of CCL28 expression in the spinal cord after SCI also possibly hint the importance of maintaining its amount at physiological level in the host.

CCL28 has been shown to serve as a chemokine attracting CCR10-expressing Treg cells and mucosal lymphocytes as well as CCR3-expressing eosinophils in many distinct inflammatory circumstances [25]. We found that compared with sham group, along with the induction of CCL28 in the spinal cord after SCI, CD4^+^CD25^+^FOXP3^+^ Treg cells were more pronouncedly recruited and their number was increased in the spinal cord as determined by FACS and immunostaining analyses. Surprisingly, the recruited Treg cells specifically upregulated expression of CCR10. Indeed, following this observation, we further demonstrated that these Treg cells were recruited to the spinal cord by CCL28-CCR10 axis, and meanwhile, its another receptor, CCR3, appeared not relevant to their recruitment. From the viewpoint of functional accessibility, we guess that this might be associated with their distinct basal levels in Treg cells present in the spinal cord, as we have shown that CCR10 was more abundantly expressed than CCR3, and this precondition probably render CCR10 more easily to reach threshold level required to exert its biological role of responding to its ligand CCL28. Coincidentally, two previous studies have also shown that CCL28 facilitates the trafficking and extravasation of Treg cells through binding to CCR10 [21, 22], together with our findings, suggesting that CCL28-CCR10 axis might be a general paradigm in which CCL28 recruits Treg cells. However, the molecular mechanisms underlying the biased choice of receptor for CCL28 in Treg cells under these experimental conditions are still unknown. Deeper investigations on addressing how the expression of CCR10 and CCR3 is differentially regulated in Treg cells in the spinal cord in this context might be helpful to advance our understanding of the Treg cell recruitment mediated by CCL28-CCR10 axis. Besides, it cannot be excluded that CCL28 upregulation presumably could recruit other CCR10- and/or CCR3-expressing immune cells to the spinal cord after SCI, for example, effector lymphocytes [48], whether this is the case and their potential effect on disease process merit further investigations.

In functional study, we found that CCL28 promoted locomotor recovery after SCI, and this beneficial effect to a great extent relied on the recruitment of Treg cells, since their disrupted recruitment or cellular depletion substantially diminished the functional role of CCL28. We subsequently showed that these recruited Treg cells served as modulatory factors to mediate immune suppression in the spinal cord after SCI, as exemplified by decreased production of proinflammatory cytokines, increased level of anti-inflammatory cytokine IL-10, and inhibited proliferation of effector T cells. These phenomena are in line with our current understanding of immunoregulatory functions of Treg cells [49], and suggest that the beneficial effect of Treg cells on locomotor recovery after SCI at least in part involves restricted local inflammatory responses, as it’s well-known that the excessive inflammation plays a crucial role in the secondary damage, and accordingly its alleviation promotes functional recovery after SCI [50]. It should be mentioned that one major limitation in this part is the insufficient interrogation of the function of effector T cells and other potential biochemical events affected by CCL28-mediated recruitment of Treg cells. Addressing these issues would help us to fully understand how CCL28 regulates the inflammatory niche in the spinal cord and promotes locomotor recovery after SCI.

## CONCLUSIONS

Our study uncovers the previously unrecognized roles of CCL28 and Treg cells recruited by CCL28-CCR10 axis in regulating the course of locomotor recovery after SCI, and associates these roles with their immunosuppressive function in local inflammatory milieu, thus highlighting the importance of targeting excessive inflammation in treating the secondary damage after SCI. Based on our findings, we propose here that manipulating the interaction between CCL28 and CCR10 might represent a novel strategy in improving functional recovery after SCI.

## MATERIALS AND METHODS

### Antibodies and reagents

The antibodies and reagents were obtained from the following sources: anti-CCL28 (Santa Cruz, sc-376654), anti-GAPDH (Abcam, ab9485), anti-phospho-NF-κB p65 (Ser536) (Cell Signaling, 3033), anti-NF-κB p65 (Cell Signaling, 8242), anti-phospho-IκBα (Ser32) (Cell Signaling, 2859), anti-IκBα (Cell Signaling, 4812), anti-FoxP3 (Novus, NB100-39002), anti-CCR10 (Invitrogen, PA1-21617), ML120B (Sigma, SML1174). Goat anti-rabbit IgG-HRP (Abcam, ab6721), goat anti-mouse IgG-HRP (Abcam, ab6789), goat anti-rabbit IgG-PE (Santa Cruz, sc-3739), goat anti-mouse IgG-FITC (Santa Cruz, sc-2010), goat anti-mouse peroxidase-conjugated IgG (Millipore, AP124P). Neutralizing antibodies against mouse IL-1β (AF401), TNF-α (AF410), CCL28 (MAB533), CCR10 (MAB2815), CCR3 (MAB1551), CD25 (AF2438) and IgG isotype control (43414), and recombinant mouse CCL28 (533-VI) were purchased from R&D Systems.

### Mouse SCI model

The mouse SCI model was established as previously described [51]. Briefly, male C57BL/6J mice of 12 weeks of age were anesthetized with 330 mg/kg of 2-2-2 Tribromoethanol (Sigma) intraperitoneally and undertook a laminectomy at the T12 vertebrae. Then a contusion SCI (hemitransection) was induced on the exposed dorsal surface of dura mater through using a SCI micro-scissor devise (Infinite Horizon impactor at 60 kDyn, Precision Systems and Instrumentation, USA). Sham group includes the complete procedures without hemitransection of the spine. During recovery, mice were maintained in an isothermic cage. For animal welfare, all animal experimental procedures were approved by the Ethics Committee of the third hospital, Hebei Medical University.

### Intraspinal injection of neutralizing antibodies and recombinant CCL28

Mice were injected with neutralizing antibodies or mouse recombinant CCL28 into intraspinal region 12 hrs before sham or SCI injury, as adapted to previous description [52]. For recording the locomotor recovery of mice, the intraspinal region was repeated at 14 days after SCI induction. In brief, 4 μl neutralizing or isotype IgG antibodies (10 ng/μl, diluted in 1% mouse serum), 4 μl mouse recombinant CCL28 (rMCCL28, 25 ng/μl, diluted in 1% mouse serum) or 1% mouse serum were injected into the uninjured spinal cord of mice (n=8 mice in each group).

### Immunofluorescence

Mice were subjected to sham or SCI surgery, and 6 hrs, 12 hrs and 14 days after injury, the transverse sections 3 mm rostral to the injury epicenter were obtained. Immunofluorescent staining was conducted as previously described [53]. In brief, the harvested spinal cord tissues were fixed overnight at 4 °C, and then transferred sequentially into 10% and 30% sucrose and immersed overnight. The spinal cord tissues were subjected to fast frozen in isopentane. Sections with 10 μm thick were prepared and blocked with 5% BSA for 1 hr, and then incubated with primary antibodies against FoxP3 (1:200) or CCR10 (1:100) overnight at 4 °C. Sections were rinsed with PBS and incubated with secondary antibodies (FITC-conjugated goat anti-mouse IgG or PE-conjugated goat anti-rabbit IgG, 1:1000) for 1 hr at room temperature in the darkness. After staining and rinse, sections were mounted with ProLong Anti-Fade reagent (Molecular Probes) and imaged under a Zeiss LSM510 laser scanning confocal microscope.

### Locomotor function assessment

Open-field locomotion was used to assess the recovery of motor function after SCI. Each group contains 8 mice and all mice were evaluated for functional recovery. Each mouse was examined every two days in the first week and weekly thereafter until 4 weeks. Motor function of the hind-limbs was measured by the locomotor rating test based on the nine-point Basso Mouse Scale for Locomotion (BMS) scale, as described in detail in a previous literature [54]. All tests and following data analysis were performed double-blindly by 3 investigators blinded to experimental condition. The final scores are presented as mean ± SEM.

### Western blot analysis and ELISA assay

Nearly 5-mm size spinal cord tissue containing the injured region were collected from 5 mice of each group at indicated time points after SCI. Samples were completely homogenized with RIPA lysis buffer (Beyotime, P0013D) supplemented with 1% protease inhibitor (Roche) on ice for 20 min. Cell lysates were centrifuged at 12000 × g for 10 min at 4 °C to collect supernatants. The protein concentration was determined by BCA kit (Beyotime) and followed by denature at 100 °C for 5 min in 1 × SDS loading buffer. Western blot was conducted as described in previous study [55]. Briefly, samples with equal amount of proteins were loaded and applied to 10% SDS-PAGE and then transferred onto nitrocellulose membranes (Millipore). The membranes were blocked and then incubated with the specific primary and secondary antibodies. Protein bands were visualized with ECL detection reagent (ThermoFisher Scientific) and quantified using ImageJ software. Cytokine concentration was quantified with the mouse Quantikine ELISA kits (R&D Systems) according to the manufacturer’s instructions for detecting mouse CCL28, IL-1β, TNF-α, IL-6 and IL-10 in spinal cord supernatants. Each group contains 5 mice. Each sample from one mouse was measured in quintuple and results are presented as mean ± SD.

### RNA extraction and RT-qPCR analysis

Spinal cord samples in each group were collected as mentioned above, and were immediately stored at −80 °C for further total RNA extraction. The total mRNA was extracted using the RNeasy Kit (Qiagen), and 2 μg total RNA were reverted into cDNA using the MultiScribe™ MuLV (ThermoFisher Scientific) according to the manufacturer’s instructions. Quantification of mRNA levels of target genes was accomplished using the SYBR Green PCR Master Mix (Invitrogen) and ABI 7300 instrument (Applied Biosystems). The mRNA level of genes was determined with the comparative threshold cycle method. Mouse *gapdh* was used as a normalization. The primers used in this study are listed as follows: Ccl28 forward 5′-CCACCGCACTTGACTCTAGA-3′, reverse 5′- CTCACACCCTGAAAACCTGC-3′; Gapdh forward 5′-CCATGGAGAAGGCCGGGG-3′, reverse 5′-CAAAGTTGTCATGGATGACC-3′.

### Flow cytometry

Spinal cord samples were collected from each group as mentioned above. Samples were dounced into single-cell suspensions using 70-μm cell strainer (BD). Live cells were obtained with Percoll gradient isolation and washed with PBS and incubated for 30 min on ice with antibodies if surface markers were detected. For intracellular staining, cells were fixed and permeabilized prior to stained with antibodies. Antibodies are listed as follows: APC-Cy7-anti-CD3 (BD Pharmingen), PE-anti-CD4 (BD Pharmingen), PE-Cy7-anti-CD25 (BD Pharmingen), APC-anti-FOXP3 (eBioscience, FJK-16s), APC-anti-CCR10 (R&D Systems, 248918) and PerCP-anti-CCR3 (R&D Systems, 83101). Flow cytometry was conducted with LSR II instrument (BD Biosciences) and data analysis was performed with Flowjo software (Treestar).

### Incorporation of [^3^H]-thymidine

The CD3^+^FOXP3^-^ (effector) T cells isolated from the spinal cord were cultured *in vitro* in the 96-well microtiter plates (Nunc) containing X-VIVO medium (Lonza) supplemented with 10% FBS, with a density of 2 × 10^5^ cells per well in triplicates. The [^3^H]-thymidine (Perkin Elmer) was added into the medium for a final concentration of 0.2 MBq/ml. Cells were further cultured for 16 hrs and the ^3^H activity was evaluated by the liquid scintillation counting (Perkin Elmer).

### *In vitro* transwell migration assay

Mouse peripheral blood mononuclear cells (PBMCs) were isolated, resuspended and one million cells were seeded in the upper migration chambers containing DMEM supplemented with 10% FBS, 100 U/ml penicillin, 100 μg/ml streptomycin, and 10 mM glutamine. Cells were pretreated with isotype control antibody or neutralizing antibodies against CCL28, CCR10 or CCR3 for 1 hr. The migration activity of cells was examined by a modified Boyden chamber assay for 4 hrs incubation with recombinant mouse CCL28 or 1% mouse control serum. The percentages of CD4^+^CD25^+^FOXP3^+^ Treg cells among the CD4^+^ cells recruited to the lower chambers were analyzed by flow cytometry.

### Statistical analysis

All data are expressed as mean ± SD, except for the BBB scores, which are expressed as mean ± SEM. Statistical analysis was performed using SPSS 17.0 (SPSS Inc., Chicago, IL, USA). Unpaired Student *t*-test was used to calculate statistical significance, and BBB scores were analyzed by repeated measures analysis of variance (ANOVA). A *p* value less than 0.05 is considered to be statistically significant.

### Ethics approval

All animal treatments were reviewed and approved in advance by the Ethics Committee of the third hospital, Hebei Medical University.
